# An evaluation of traffic-awareness campaign videos: empathy induction is associated with brain function within superior temporal sulcus

**DOI:** 10.1186/1744-9081-10-27

**Published:** 2014-08-12

**Authors:** Jana Zelinková, Daniel J Shaw, Radek Mareček, Michal Mikl, Tomáš Urbánek, Darina Havlíčková, Petr Zámečník, Petra Haitová, Milan Brázdil

**Affiliations:** 1 Behavioral and Social Neuroscience Research Group, CEITEC - Central European Institute of Technology, Masaryk University, Brno, Czech Republic; 2 First Department of Neurology, Masaryk University, St. Anne’s Faculty Hospital, Pekařská 53, Brno 656 91, Czech Republic; 3 Institute of Psychology, Academy of Sciences of the Czech Republic, Veveří 97, Brno 602 00, Czech Republic; 4 Traffic Psychology Department, Transport Research Center, Vinohrady 10, Brno 639 00, Czech Republic

**Keywords:** fMRI, Traffic-awareness campaign, STS, Socio-emotional processing

## Abstract

Acting appropriately within social contexts requires an ability to appreciate others’ mental and emotional states. Indeed, some campaign programs designed to reduce anti-social behaviour seek to elicit empathy for the victims. The effectiveness of these campaigns can be evaluated according to the degree to which they induce such responses, but by applying neuroscientific techniques this can be done at the behavioural *and* neurophysiological level. Neuroimaging studies aimed at identifying the neural mechanisms behind such socio-cognitive and -emotional processes frequently reveal the role of the superior temporal sulcus (STS). We applied this knowledge to assess the effectiveness of traffic-awareness campaign adverts to induce empathic expression. Functional magnetic resonance imaging (fMRI) data were acquired from 20 healthy male volunteers as they watched these campaign videos consisting of a dramatic sequence of events and catastrophic endings, and control videos without such dramatic endings. Among other structures, a significantly greater neural response was observed within bilateral STS, particularly within the right hemisphere, during the observation of campaign relative to control videos. Furthermore, activation in these brain regions correlated with the subjects’ empathic expression. Our results develop our understanding of the role of STS in social cognition. Moreover, our data demonstrate the utility of neuroscientific methods when evaluating the effectiveness of campaign videos in terms of their ability to elicit empathic responses. Our study also demonstrates the utility of these specific stimuli for future neuroscientific research.

## Introduction

Recent research employing functional magnetic resonance imaging (fMRI) has begun to reveal that the same brain systems involved in socio-cognitive and -emotional processes are implicated also in pro-social behaviour [1,2]. Applying these findings will allow us to develop and evaluate campaign programs aimed at reducing anti-social behaviour, both at the behavioural *and* neurophysiological level; an effective campaign should elicit pro-social behavioural responses and brain function within associated neural systems. In this light, we can not only assess the effectiveness of a program to elicit the desired response, but also identify the mechanisms through which it exerts this effect. The present paper describes a study that attempted exactly this with a set of traffic-awareness campaign videos.

Anti-social behaviour – e.g. aggression and a lack of concern for others – often occurs in driving situations, presenting a potential danger to all drivers. In 2012, the European Commission for Mobility and Transport reported over 27,000 fatalities from road-traffic accidents throughout Europe alone. For this reason, societies try to prevent antisocial driving behaviour by means of traffic-awareness campaigns, the aim of which is to motivate drivers to avoid endangering themselves and others. One such campaign was broadcasted recently throughout the Czech Republic: *Nemyslíš Zaplatíš (If you don’t think, you will pay)*. This particular campaign attempts to alter attitudes towards dangerous driving by highlighting the negative consequences to others. More specifically, following the principles of empathy induction [3,4], this campaign attempts to elicit empathic and compassionate reactions towards the victims of various road-traffic accidents. Although national statistics show that the number of traffic-related deaths is decreasing yearly, this declining trend was described before the televised campaign began. It is unclear, therefore, if and how these campaign videos affected people in the intended manner. Answers to this question have important implications for the field of social and behavioural neuroscience in general; namely, how do individuals respond behaviourally to campaign programs that follow the principle of empathy induction, and what neural systems underlie this response?

The aim of our study was to examine whether these campaign videos - developed to induce empathy - do indeed elicit this socio-emotional reaction and brain function within neural systems underlying such a response. If they are successful at inducing empathy, we hypothesised that this would manifest as greater empathic expression when individuals describe the video content. We also hypothesised that the videos would elicit brain function within certain structures. Research suggests the superior temporal sulcus (STS), for example, plays a crucial role in such socio-emotional processing; in particular, brain function within STS is engaged consistently during tasks that require us to infer and share in another individual’s mental e.g. [5-7] and emotional state e.g. [8,9] – i.e. mentalising and empathy, respectively. Given this role of STS, we expected brain function within this region to be related to individuals’ emotional and empathic reactions to the stimuli.

To investigate this we developed an fMRI experimental design with which we compared brain function during the campaign video clips with that elicited by control videos. The former consisted of anti-social driving behaviour together with various catastrophic consequences, while the latter presented analogous driving themes without dramatic endings. To measure behavioural responses to the campaign stimuli, we performed thematic analyses of individuals’ subjective evaluations, creating various indices of socio-cognitive and -emotional reactions. Importantly, these various indices were designed to capture both the cognitive and affective components of empathy. We then assessed the extent to which brain function elicited within associated neural systems during the campaign videos was related to these behavioural indices.

As part of an ongoing study into dangerous driving, the present investigation was conducted on a group of male drivers who will serve eventually as a matched control group for dangerous drivers. We present only the data from this control group as means of exploring the behavioural and neural effects of these campaign videos on typical drivers.

## Methods

### Subjects

As part of an on-going investigation into driving behaviour, we recruited 20 healthy right-handed male volunteer drivers who reported no traffic offenses or traffic accidents. The mean age was 22.1 yrs (SD = ±2.1 yrs; range = 20-28 yrs; median = 22 yrs). All participants were experienced drivers, possessing a driving license for at least several months (median = 3 yrs; maximum = 10 yrs) and driving at least once every other month (median = 1/week). All had normal or corrected-to-normal vision, and spoke Czech or Slovak as their first language. Written informed consent was obtained from each subject prior to the experiment, and the study received the approval of St. Anne’s Hospital Ethics Committee.

### Stimuli

During the scanning procedure, subjects viewed a series of twelve 30-second video clips representing various types of driving situations. Six clips were taken from a national traffic-awareness campaign – *Nemyslíš Zaplatíš* (campaign videos [CV]). Each involved a dramatic sequence of events (e.g. risky overtaking) that implied and resulted ultimately in a catastrophic and tragic ending (e.g. resuscitation, death), illustrating various potential consequences of traffic accidents. These video clips, broadcast widely throughout Czech Republic between 2008 and 2010, were prepared by a professional agency in cooperation with the Ministry of Transport. This campaign was targeted especially at young male drivers and the most common causes of traffic accidents, including high-speed driving, alcohol consumption, and driver aggression. These CV stimuli were presented pseudo-randomly (see below) with six control videos (neutral videos [NV]). These NV stimuli were created in our lab by extracting sequences from typical car advertisements involving various traffic situations, and followed analogous driving themes but lacking any undertones of danger and without tragic endings. All CV and NV clips contained sound, presented binaurally via MRI-compatible headphones. All clips contained a very similar number of performers and lasted identical durations. Overall, the CV and NV stimuli were equivalent in terms of the number of individuals involved and interactions among them.

No more than two instances of the same stimulus category (CV or NV) succeeded one another. A 26-second pause was inserted between the videos, consisting of a central yellow cross against a black background. Visual stimuli were shown via a back-projection screen onto an overhead mirror. The subjects were instructed to remain still while in the scanner and to watch passively the presented video clips. Subjects were informed that some clips would have a dramatic end, but they should attend closely to the stimuli throughout. Subsequent behavioural examination (below) confirmed that all participants attended closely to all instances of both CV and NV stimuli.

### Magnetic resonance imaging (MRI): acquisition parameters

Imaging was performed on a 1.5 T Siemens Symphony scanner equipped with Numaris 4 System (MRease). The functional scans were obtained using a gradient echo, echoplanar imaging sequence: TR = 3000 msec, TE = 40 msec; FOV = 220 mm; flip angle = 90°; matrix size 64 × 64, slice thickness = 3.5 mm; 32 transversal slices per scan. Functional measurement consisted of 220 scans. The imaged volume covered most of the brain, excluding the vertex. Following functional measurements, high-resolution anatomical T1-weighted images were acquired using a 3D sequence that served as a matrix for the functional imaging (160 sagittal slices, resolution 256 × 256 resampled to 512 × 512, slice thickness = 1.17 mm, TR = 1700 msec, TE = 3.96 msec, FOV = 246 mm, flip angle = 15°).

### Behavioural examination

Immediately after MRI scanning all subjects completed a short questionnaire concerning their previous knowledge of the campaign clips. This confirmed that the CV and NV stimuli were equally familiar to our sample. During this post-scanning session, each subject was shown individually all of the campaign stimuli again. To investigate emotional reactions they were asked to evaluate each of them in terms of valence and arousal ratings, on a scale from 1 (pleasant/peaceful) to 10 (unpleasant/exciting). To examine socio-cognitive and -emotional processes elicited by the stimuli, subjects were asked to provide a time-unlimited verbal description of the content of each campaign video clip.

We categorized the descriptions of the CV stimuli by analysing their thematic content. Assuming that participants would describe those aspects that they attended to most closely and processes most deeply, we creating special indices that represented distinct components of social cognitive and emotional processing: (1) Meta-cognitive awareness of the purpose of the campaign; (2) subjects’ interpretations of situational aspects (e.g. relationships, car functionality); (3) subjects’ evaluation of the campaign videos; (4) subjects’ self-referencing; (5) subjects’ awareness of their own mental state and (6) emotional reactions during the stimuli; (7) subjects’ awareness of the video actors’ mental and (8) emotional states; (9) subjects’ awareness of the consequences of characters’ actions; and (10) incidences of subjects’ perspective-taking.

The descriptive texts were then divided into short utterances that were scored according to the selected thematic content. In this way, we obtained frequencies for particular thematic categories that then formed the aforementioned indices. To avoid any biases in the analysis, verbal fluency – i.e. the total number of words spoken – was taken into account; specifically, the frequencies for each index were divided by the number of words produced by the participant [10]. In subsequent analyses, non-parametric Spearman correlations were employed since not all indices conformed to a parametric distribution.

Since indices 7, 8, 9 and 10 were designed to measure socio-cognitive and -emotional processing, we summed the respective scores to create one “empathic expression” index. Importantly, the constituent indices were correlated positively with one another (range: r = .39 – .89) and negatively with those designed to measure more self-focused and egocentric processing (i.e. indices 3, 4, 5 and 6; range: r = −.22 – -.66).

### Functional MRI analyses

The functional and structural MRI data were analysed using SPM5 (Functional Imaging Laboratory, Wellcome Department of Imaging Neuroscience, Institute of Neurology, University College London, UK) running under MATLAB 7.6 (Mathworks Inc., USA). The following pre-processing steps were applied to each individual’s functional time series: (1) realignment to correct for any motion artefacts, (2) normalization to fit into a standard anatomical space (MNI) using parameters derived from high-resolution anatomical T1-weighted image, (3) spatial smoothing using a Gaussian filter with a FWHM of 8 mm, (4) high-pass filter with a cut-off at 256 sec, and (5) an autoregressive model to estimate serial autocorrelations. The resulting pre-processed functional images were resampled to a resolution of 3 × 3 × 3 mm.

A General Linear Model (GLM) was implemented in SPM5 to identify whether any brain regions expressed greater blood oxygenation level-dependent (BOLD) signal during either of the two active conditions (CV or NV) relative to the fixation baseline. The experimental stimulation time-course for each condition was convolved with a canonical hemodynamic response function. In addition, 6 time series of movement parameters derived during realignment of the fMRI scans were added to the GLM to regress out residual effects of head movement. Statistical parametric maps with t-statistics were computed to assess the significance of BOLD signal increases during the CV or NV condition relative to baseline, and to assess differences in BOLD signal between the two conditions. Corresponding contrast files were used for the second-level analysis. We used a random-effect analysis (one-sample *t*-test) to assess the mean group difference between CV and NV conditions. For this whole-brain analysis, the significance level was set to p < 0.05, corrected for multiple comparisons by controlling the Family Wise Error (FWE), using Random Field Theory. The extent threshold was set to 5 voxels. Subsequently, spheres of 5 mm radius centred at the cluster peaks emerging from this CV > NV comparison served as regions of interest (ROIs), within which we examined relationships between BOLD signal and our behavioural measures of empathy, valence and arousal.

To assess whether emotional reactions to the CV stimuli were related to brain function, we investigated if clip-specific valence and arousal had a modulatory effect on the BOLD signal amplitude. Given our primary focus on brain systems involved in socio-emotional processing, we explored this potential for modulation within the same ROIs expressing greater BOLD signal during CV relative to NV (above). For each subject, valence and arousal ratings were adjusted to have a zeroed mean and unitary standard deviation. We then computed the first eigenvector from the time-series of all voxels comprising a given ROI, adjusted for the effects of interest. This was used as a representative ROI/subject-specific BOLD signal. Two GLMs were set and estimated for the CV condition, and valence or arousal entered as a parametric modulator. Two one-sample t-tests were then used to assess the group effects of valence and arousal parametric modulators on CV video category. Significance was set at p < 0.05. In light of our strong *a priori* hypotheses concerning the relationship between brain function within STS and socio-emotional processing, and since only one ROI lay outside of this brain structure (see Table 1), multiple-comparison correction was not performed on these ROI-based analyses. Two subjects were discarded from this analysis, as one had rated all NV clips equally, and the other had rated all CV clips equally.

**Table 1:** Brain regions in which BOLD signal was significantly greater (p < 0.05, FWE corrected) during campaign vs. neutral videos (CV > NV contrast)

**ROI index**	**Region**	**MNI Co-ordinate (mm)**	**Z max**	**# Voxels**
1	L STG/STS/MTG	−60, −9, 3	5.74	87
		−60, −26, 6	5.28	
2	R STG/MTG	60, 6, −12	5.38	12
3	L IPL	−63, −39, −27	5.22	12
4	L MTG	−45, −60, 12	5.20	10
5	R MTG	63, −9, −6	5.13	10
6	R MTG	63, −21, −3	5.02	21
		51, −20, −6	4.95	
7	R STS	51, −42, 9	4.96	9

NB: These clusters served as regions of interest in subsequent analyses. *Abbreviations:L* left, *R* right, *STG* superior temporal gyrus, *STS* superior temporal sulcus, *MTG* middle temporal gyrus, *IPL* inferior parietal lobule.

We then investigated if the BOLD signal increase during the CV relative to the NV condition was modulated by empathy. Unfortunately, three subjects were removed from these analyses because the recording of their verbal description was unavailable. For the remaining 17 subjects, the mean CV-NV differences were drawn from the respective contrast files within each of the same ROIs defined above, and non-parametric Spearman correlation was then used to evaluate if a relationship existed between BOLD signal and empathic expression. For the same reasons given above, multiple-comparison correction was not applied to these ROI analyses.

## Results

**Figure 1:**
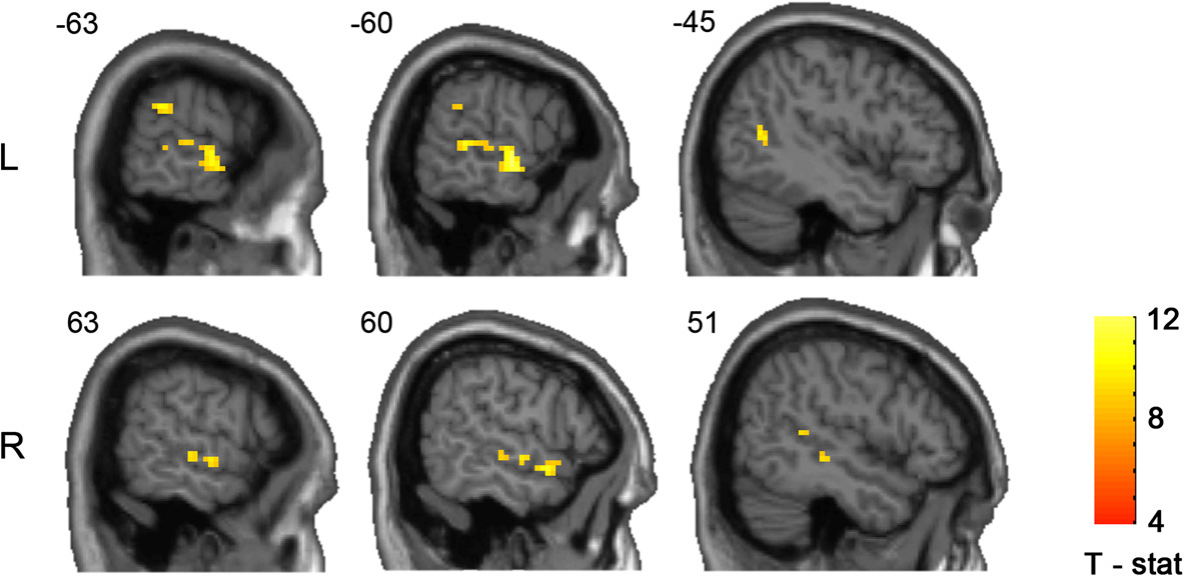
**Brain areas with significantly greater BOLD signal during campaign compared with neutral videos.** Image is thresholded at p < 0.05, FWE corrected, spatial extent threshold = 5 voxels. *R* = right, *L* = left.

We analysed the main effects of stimulus type on brain function by contrasting BOLD responses to the campaign (CV) and neutral video clips (NV). The maximum significant difference in neuronal activity was observed within bilateral superior temporal sulci, particularly within the left hemisphere (Figure 1). The BOLD signal within this brain region was greater during CV compared with NV. Other, less extensive differences in this direction were observed in the left inferior parietal lobule (IPL). The results of this whole-brain analysis are summarized in Table 1. Spheres centred on these seven peak voxels from within these significant clusters served as our ROIs in the subsequent analyses.

As expected, the subjective evaluation of valence and arousal differed between CV and NV: campaign videos were evaluated as more unpleasant and arousing than the control stimuli (Table 2). When arousal ratings were entered as parametric modulators, we observed no significant effects of valence or arousal ratings on BOLD signal in any ROI. This suggests no relationship between subjective emotional evaluations of the campaign stimuli and BOLD signal within brain region activated more during the campaign relative to the control stimuli.

**Table 2:** Average values of subjective emotional ratings of valence and arousal for campaign (CV) and neutral videos (NV)

**Affective dimension**	**Video category**	**Subjective evaluation**
Valence	CV	7.25 (±2.41)
	NV	3.05 (±1.87)
Arousal	CV	7.06 (±2.43)
	NV	3.05 (±1.88)

**Figure 2:**
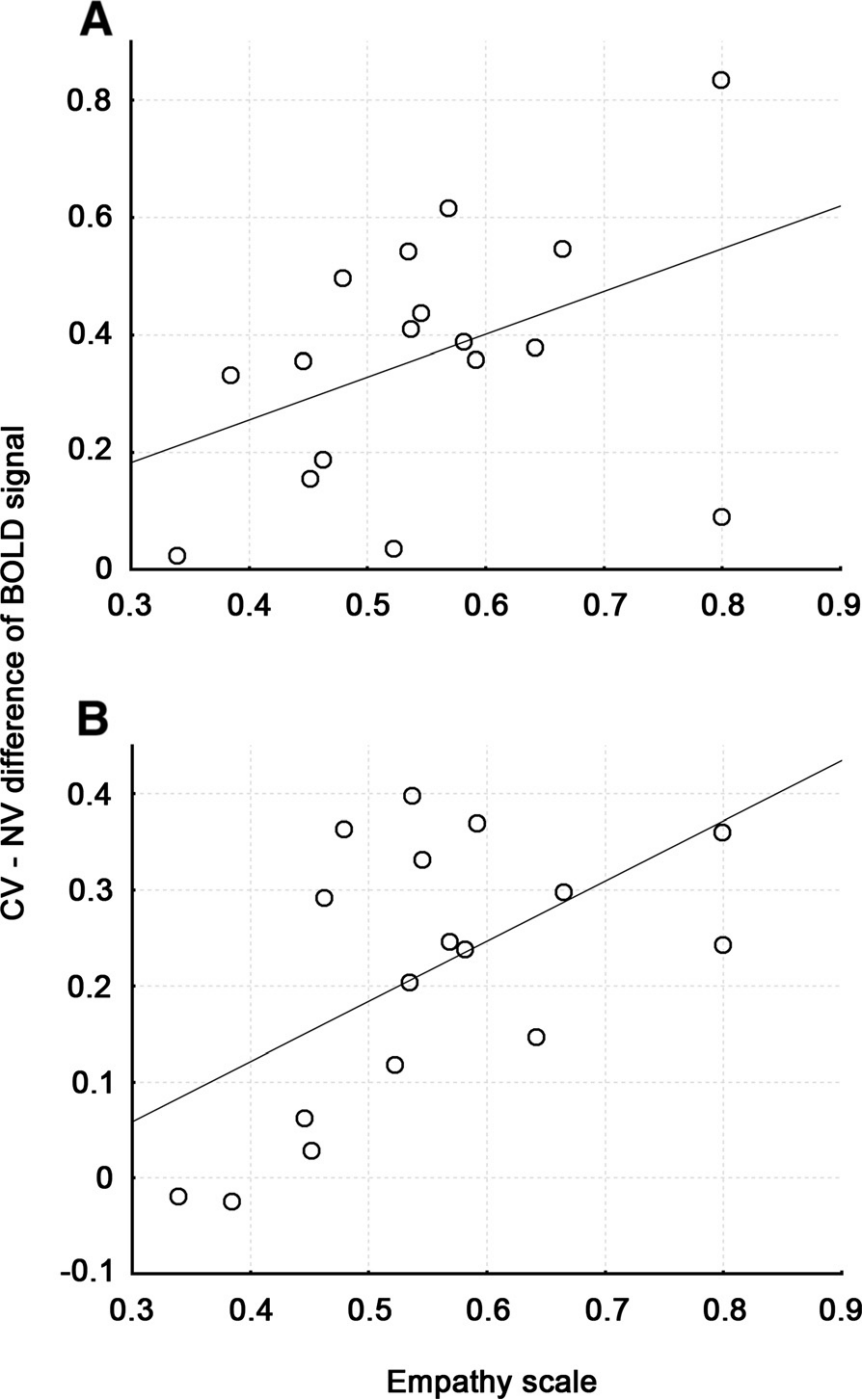
**Relationship between empathic expression and increased BOLD signal in the CV > NV contrast within (A) right MTG (ROI-5, 63, −9, −6; r = 0.478; p = 0.052, uncorrected) and (B) right STS (ROI-7, 51, −42, 9; r = 0.551; p = 0.022, uncorrected).**

Given our hypothesis concerning the role of STS in socio-emotional processing, we investigated the relationship between our composite behavioural measure of empathic expression and the CV > NV difference in BOLD signal within ROIs encompassing this brain structure. This revealed that greater brain activation during the campaign stimuli within right STS was related to higher empathic expression. This is illustrated in Figure 2 and Table 3. These correlations reveal an interesting and meaningful positive relationship between the response of STS during these social stimuli and socio-emotional reactions.

**Table 3:** Trend in relationship between greater BOLD signal in the CV > NV contrast, and empathic expression

**ROI index**	**Region**	Correlation*	***p*** **value (uncorrected)**
1	L STG/STS/MTG	.309	.228
2	R STG/MTG	.196	.451
7	R STS	.551	.022

*Abbreviations: R* right, *L* left, *STS* superior temporal sulcus, *STG* superior temporal gyrus, *MTG* middle temporal gyrus. *= Spearman’s Rho co-efficient. Uncorrected probability values are given.

## Discussion

The purpose of this study was to evaluate whether a set of traffic-awareness campaign videos developed to induce empathy are capable of eliciting such behavioural socio-emotional reactions, and neural activity within associated brain areas. To do so, we first compared brain function during the observation of campaign videos (CV) with control video clips (NV), the latter containing analogous driving themes but without behaviour that endangers others and no emotional endings. We then examined whether or not subjective emotional ratings and a measure of empathic expression modulated the differences in brain function elicited by the campaign stimuli. Two primary findings emerged.

Firstly, we found that the CV elicited greater hemodynamic response than the NV within bilateral STS and STG. A wealth of evidence demonstrates a connection between brain function within STS and socio-cognitive processing e.g. [11-14]. Further, an increasing number of studies report a link between STS dysfunction in autism [15,16] – a disorder characterised in part by deficits in social cognition – and the pathogenesis of psychopathy [17]. Finally, [18] revealed that observing others’ risk-taking behaviour elicited brain activity in a number of brain regions that included STG. Such findings are consistent with Peelen et al.’s suggestion that the STS plays a key role in understanding and categorizing the mental and emotional states of others [7]. Our analyses also revealed the involvement of IPL. Interestingly, this latter structure hosts extensive reciprocal connections with a considerable portion of STS [19]. Moreover, both STS and IPL have been described in connection with processes central to social cognition (e.g., eye-gaze processing [20]).

Since empathy provides an affective and motivational basis for compassion and altruism (e.g. [1], see also [21]), this implicates the STS in pro-social behaviour. Consistent with this notion, atypical empathy-related neural responses within STS are reported in individuals with anti-social proclivities [9,17,22]. This indicates that our data reveal the role of STS in the cognitive and emotional evaluation of others’ social behaviour. Interestingly, during their verbal descriptions, our participants frequently reported sympathy with the depicted victims of traffic accidents. The ability of these campaign stimuli to elicit such behavioural and neurophysiological responses suggests the potential effectiveness of empathy training as an intervention for anti-social driving behaviour. This, in turn, suggests similar neuroimaging evaluations can be conducted also for some intervention programs (e.g. Compassion Training; [23,24]; for reviews see [3,4]).

The second primary finding is that, using these novel social stimuli, we also demonstrate a relationship between socio-emotional processing and brain function within right STS; namely, increased BOLD signal during CV relative to NV clips was greater in individuals who engaged in greater empathic expression. In the last decade, many studies have been conducted to determine how the human brain mediates social cognitive and emotional processes. Multiple studies have revealed associated neuronal activity in the anterior paracingulate cortex, the STS, and the temporal poles bilaterally. As such, these areas are considered generally to be central to social behaviour and social cognition [5].

The effectiveness of the campaign stimuli in eliciting socio-cognitive/-emotional reactions and associated brain function provides an important methodological contribution to the field of social cognitive neuroscience; in particular, we present novel, socially rich stimuli that overcome many of criticisms of the artificial and uni-dimensional nature of stimuli employed typically. Traditionally, neuroimaging studies have explored neural systems involved in social cognitive and emotional processing by examining brain function during highly artificial stimuli designed to elicit single cognitive processes, such as emotional faces e.g. [25] or bodies e.g. [26]. More recently, Lahnakoski et al. [27] demonstrated within a single neuroimaging experiment the role of STS during the processing of a wide variety of social cues (e.g. biological motion, faces, social interactions, speech) [27]. As the authors point out, this demonstrates the potential for fMRI mapping under conditions that resemble more accurately the complexity of real-life social contexts. Coincidently, Zaki and Ochsner evaluate critically the use of artificial stimuli that differ qualitatively from typical social inputs, and a lack of consideration for relationships between brain activity and social behaviour [28]. Together, this illustrates the need to move away from studying single socio-cognitive and emotional processes with narrow categories of social stimuli (e.g. perception of faces vs. bodies), towards comparisons between more complex social stimuli. By integrating a wide variety of social cues and eliciting a range of cognitive and emotional processes (e.g. mentalising, empathy, moral judgment), we suggest that the *Nemyslíš Zaplatíš* campaign videos provide useful stimuli for social neuroscience research.

It is important to acknowledge some potential limitations in our experimental design. First, we are aware of the potentially circular nature of our analytical approach – that is, extracting the relative increase in BOLD signal measured during the campaign stimuli within STS and correlating it with the composite empathic expression score created from subjective reports. Such an approach could be considered a circular analysis [29,30]. This would overestimate the relationship between brain function and indices of socio-emotional processing [31]. It is suggested that circularity can be avoided by interrogating anatomically defined regions of interest [29]. Unfortunately, employing such an approach seemed to dilute any relationships we observed between brain function in STS and empathic expression. This is likely to due to the relatively large size of the superior temporal gyrus, containing a large number of voxels. Importantly, however, the relationship between brain function and our composite measure of empathic expression was not observed throughout all data-driven regions, and increased brain function within STS was not correlated with individual indices of social cognition. Furthermore, as we have summarised above, our results are meaningful theoretically and in light of previous empirical research.

We are also aware that the results of our ROI analyses would not survive stringent multiple-comparison correction. Although our focused, hypothesis-driven analyses justified this approach, it is possible that these results reflect a type I error arising from the number of comparisons conducted, rather than legitimate brain-behaviour relationships. We believe it is more likely that the relatively modest effects we observed result instead from the procedure we employed: Firstly, subjects in the present study were not asked to engage in any explicit emotional processing during fMRI scanning; second, affective reactions were provided upon seeing the videos for a second time, after fMRI scanning. Alternatively, it is possible that revealing any influence of emotion processing on BOLD signal requires a sample size greater than that used in the present study.

Finally, it is important to acknowledge the limitation of the study in terms of the all-male sample employed, since this clearly limits the generalizability of our results. We present the data from a group of drivers that will serve eventually as a control group in our ongoing research into dangerous driving. Due to the fact that recidivist offenders of driving violations are predominantly male e.g. [32,33], our final sample will necessarily be male dominated. Moreover, since dangerous drivers are predominantly male, we consider it very important to understand the socio-cognitive and emotional processing undertaken by less anti-social male drivers. Indeed, despite these potential shortcomings, our data not only provides strong evidence for a connection between the STS region and socio-cognitive/-emotional processing, but also demonstrates that driving is inherently a social action. This should be emphasized to future drivers during their training.

## Conclusions

In this study we have revealed that traffic-awareness campaign videos, depicting socially dangerous behaviour ending with tragic consequences, engage the STS preferentially. Moreover, we observed a relationship between the expression of empathy towards the victims of dangerous driving and brain function within right STS. Our findings, then, not only provide an evaluation of these campaign videos according to their ability to elicit behavioural and neurophysiological indices of empathic expression; they also confirm the role of the STS in high-level social cognitive and emotional processes using novel, complex social stimuli. The STS seems be a central node of brain networks supporting social cognition in general, and empathy in particular. Of course, it likely that the STS support multiple cognitive operations depending on task-dependent network connections, or that it is involved in more elementary low-level processes that together contribute to social behaviour. These findings raise questions that can be addressed in future work, and we suggest that our campaign stimuli will prove very beneficial in this endeavour.

## Competing interests

The authors declare that they have no competing interests.

## Authors’ contributions

JZ is the main author, she carried out interactions with participants and composed the outputs. DJS is the English speaking consultant and co-worker on the editing of the manuscript. RM and MM are engineers, they ensured fMRI data and technical procedure. TU is the psychological consultant, he is co-worker on behavioral data measurement and outputs. DH and PZ are specialists from traffic research, they ensured recidivist volunteers. PH is co-worker due to her knowledge of STS functions. MB is the main supervisor of the study and all-sections consultant. All authors read and approved the final manuscript.
